# A Single Center Review of the Dangers of Recreational Fires in the Pediatric Population

**DOI:** 10.1093/jbcr/iraa095

**Published:** 2020-11-17

**Authors:** Vinu Perinjelil, Robert Stephen Haake, Afroze Ahmed, Fadi Al-Daoud, Tareq Maraqa, Leo Mercer, Kristoffer Wong, Stephen Morris, Donald Scholten, Gul Sachwani-Daswani

**Affiliations:** 1 Hurley Medical Center, Flint, Michigan; 2 Michigan State University, East Lansing, Michigan; 3 Henry Ford Wyandotte Hospital, Wyandotte, Michigan

## Abstract

The increasing trend of admissions due to recreational fires prompted a 5-year review. The retrospective chart review of pediatric burn injuries from campfires or bonfires treated at a single medical center’s burn unit. The study included children within the ages of 0 to 15 admitted or transferred from January 2012 to December 2016 with first, second, and/or third degree burns by bonfires. These patients accrued burns due to active fires as well as postfire ember contact. Two hundred-eighty nine (289) were pediatric admissions out of which 66 (22.8%) were pediatric admissions associated with recreational fires. The mean annual admission for campfire or bonfire burns was 13 ± .98. The mean age was 4 ± 2.47 years. Gender distribution revealed 21 female and 45 male pediatric patients under the age of 15. From the available data, 8 (12%) of these burns occurred at home in the backyard and 16 (24%) at a public camp or park. Injury mechanisms were more commonly a result of direct contact with hot coals and embers (65%). Falls into open flame accounted for 23% (n = 15) of injuries, and flash flames accounted for 12% of injuries (n = 8). The presence of supervision was unknown in 56%; however, lack of supervision was a factor in 14% of our study population. By gaining a better understanding of the type of injury, mechanism of injury, and the demographic of recreational fire burn victims, policy, and awareness campaigns were instituted in an effort to reduce the incidence of recreational fire burns.

For many midwesterners, campfires are nostalgic symbols of warmth symbolizing comfort and community. While a relaxing way to spend your time outdoors, recreational fires pose a real danger when poorly tended or carelessly handled. Despite guidelines for open burning set by the Michigan Department of Natural Resources,^1^ outdoor recreational fires, campfires, bonfires, and grills contribute significantly to preventable burn injuries in the pediatric population.^2–5^ The lack of parental supervision, deficiency in fire education, and lack of awareness on the dangers of embers have been associated with these types of injuries.^5,6^ Open pits, lighter fluid, and inebriated parental supervisors are commonly implicated risk factors contributing to both pediatric and adult burns.^7^ These prevalent, yet preventable injuries often necessitate medical and surgical intervention under the care of trauma services with considerable morbidity. Campfires have become a concern to the trauma and acute care surgery division of our hospital as we have noticed the increasing trend of burn admissions due to recreational fires. Since recreational fires are widespread in counties within Michigan, we seek to supplement the existing literature by elucidating the underlying risk factors and promoting policy and awareness campaigns in order to decrease burns due to recreational fires.

## METHODS

We conducted a 5-year retrospective chart review of pediatric burn injuries from campfires or bonfires treated at our American College of Surgeons (ACS) verified Level I Medical Center’s Burn Unit. Children less than 15 years of age (age range defined by ACS as pediatric patients) that were admitted or transferred from January 2012 to December 2016 with first, second, and/or third degree burns by bonfires or campfires were included in our study which was approved by the Institutional Review Board. These patients experienced burns due to active fires as well as postfire ember contact. Patients aged 15 years or older were excluded from this study. Patient charts were reviewed, and data abstraction included demographic information, epidemiologic data and clinical characteristics.

Demographic data points collected from the trauma database included age, gender, burn type, mechanism of injury, comorbidities, as well as alcohol and recreational drug involvement. The endpoint data collected consisted of degree of burn, length of stay, presence of accelerant, presence of caregivers and other children, requirement of surgical interventions, and any complications arising from the burn injury and/or surgery. Injury information was stratified by injury severity score. We present a descriptive analysis of our data. A campfire safety flier highlighting precautions and reflecting our study results was designed for distribution in the hospital and regional parks.

## RESULTS

During our study period, we had a total of 1160 admissions to our burn center. Two hundred-eighty nine (289) were pediatric admissions out of which 66 (22.8%) were pediatric admissions associated with recreational fires. The mean annual admissions per year associated with recreational fires was 13 ± .98. The age of patients ranged from 12 months old to 14 years old with a mean age of 4 ± 2.47 years (Table 1). Gender distribution revealed 21 female and 45 male pediatric patients. From the available data, 8 (12%) of these burns occurred at home in the backyard and 16 (24%) at a public camp or park. For 42 (64%) patients, the location of the incident was unknown. Hospital length of stay ranged from 1 to 20 days, with a mean of 3.29. The longest hospitalization of 20 days was associated with extensive involvement of bilateral upper and lower extremities. Longer hospitalizations were associated with 2nd to 3rd degree burns as expected, with larger TBSA involved. Three patients (4.5%) had a behavioral disorder such as attention deficit disorder or attention deficit and hyperactivity disorder; five (7.5%) of patients had an underlying anxiety disorder; two patients (3%) had obsessive compulsive disorder. The presence of supervision was unknown in 56%, however lack of supervision was a factor in 14% of our study population. Inhalation injury was not present in any of our patients. There were no mortalities noted in our study population.

**Table 1.** Basic demographics of children (n = 66) sustaining campfire burns

Age (mean ± *SD*)	4 ± 2.5
Annual admissions related to campfire or bonfire burns (mean ± *SD*)	13 ± .98
	n (%)
Male	45 (68)
Female	21 (32)
Location where injury occurred*	
At home backyard	8 (12)
At public park/camp	16 (24)
Unknown location	42 (64)
Hospital LOS^†^	3.29
Identified comorbidities	
Mild pervasive developmental disorder	1
ADD/ADHD^‡^	3
Anxiety	5
OCD^‡^	2
Mood disorders including mild bipolar I	1
Autism spectrum	1
Pyloric stenosis	1
Asthma	4
History of convulsions	1
History of skull fracture/trauma	1

*Records available reviewed.

^†^Length of stay.

^‡^Attention deficit disorder/attention deficit and hyperactivity disorder; obsessive compulsive disorder.

Injury mechanisms were more commonly a result of direct contact with hot coals and embers (65%; Table 2). This included falls or contact with coals and embers. Falls into open flame accounted for 23% (n = 15) of injuries, and flash flames accounted for 12% of injuries (n = 8). Four children sustained burns due to clothing catching on fire. Clothing was a factor in two additional children, one of whom tried to stir the fire with a stick and the other child who experienced his chair collapsing close to the campfire. Two patients were injured as a result of being pushed into the fire. Friends or siblings shoved one 9-year-old female and a 2-year-old male into the fire. Seven patients experienced burns due to the use of accelerants that ranged from gas cans and lighter fluid to bug sprays and cans with unknown substances. Forty-six patients (70%) sustained burns to less than 10% of TBSA, while 19 (29%) had burns involving 10 to 20% of TBSA. Patients had a combination of first, second, and third degree burns. Sixty-two patients suffered 2nd degree burns and 17 had 3rd degree burns. The leading body part to be injured because of recreational fires were hands (n = 42; 64%), followed by lower extremities (knee/leg; n = 38; 60%) and feet (n = 30; 50%). Fifty-three percent (n = 35) of patients had involvement of two or three sites, 27% (n = 18) had four or more sites, and 20% (n = 13) had involvement of one site. Majority of burns were treated with local debridement at bedside with subsequent wound care (n = 56; 85%). Ten patients (15%) required operative treatment requiring tangential debridement with application of split thickness skin graft.

**Table 2.** Clinical characteristics of children (n = 66) sustaining camp or bonfire injuries

Mechanism of injury	Number of burn victims (%)
Falls into flame	15 (23)
Falls into embers	43 (65)
Flash with fire accelerants	7 (11)
Flash without accelerants	1 (1)
Total	66 (100)
BSA	Number of burn victims (%)
<10%	46 (70)
<10–20%	19 (29)
>20%	1 (1)
Degree of burn*	Number of burn victims (%)
1st	3 (5)
2nd	62 (94)
3rd	17 (26)
Anatomic location	Number of burn victims (%)
Head and neck	12 (18)
Shoulder	1 (1)
Arm/forearm	35 (53)
Wrist	9 (14)
Hand	42 (64)
Thorax/abdomen	6 (9)
Back	7 (11)
Buttock	4 (6)
Knee/leg	38 (60)
Foot	33 (50)
Number of sites burned per person	Number of burn victims (%)
1	13 (20)
2 or 3	35 (53)
4 or more	18 (27)
Management	Number of burn victims (%)
Local debridement plus wound care	56 (85)
Debridement plus STSG	10 (15)

*STSG*, split thickness skin graft.

*Some suffered multiple degree burns.

## DISCUSSION

Campfires and bonfires traditionally serve as the center of outdoor gatherings in Michigan communities, but carry hazardous implications for children who are burned and scarred by these fires.^5–8^ In an effort to better understand burn injury patterns and evaluate the need for preventative and public awareness efforts, we undertook a retrospective review of thermal injuries from recreational fires at our institution. Morbidity in the pediatric population is significant irrespective of area affected, as burns not only cause physical discomfort and scarring, but also can impair function of the affected area and carries a psychological impact from the trauma.^8,9^

Our analysis revealed that injuries due to recreational fires occurred at a 2:1 ratio when comparing male to females. This is consistent with the literature.^1–16^ Although our study focused on the pediatric population, a study by Neaman et al reported that 73% of the patients reported to have sustained burns related to recreational fires were males.

Our study demonstrated that over a 5-year period, almost 23% of pediatric burn admissions resulted from recreational fires and 24% of these injuries occurred away from home. In our report, falls onto embers and seemingly day old campfires constituted a majority of burns within the pediatric population. The body parts most commonly affected are the hands and knees, most likely due to the instinct of bracing the fall with the hands and finally landing in a crawl position with the knees and anterior tibial region making contact with the embers or flames. A study by Choo et al found that out of the 33 children treated for campfire burns, 75% of them were burnt by hot ashes that were not previously extinguished.^7^ Our results reflect the trend that almost three-fourths of burn injuries from recreational fires result from embers, which are assumed to be cold.

Improper methods of extinguishing recreational fires can exacerbate larger fires or injuries. Water is the best way to reduce the temperature to safe levels within 15 minutes.^2^ A retrospective review of 30 thermal contact burn patients reported in a study by Antonoff et al documented 25 pediatric injuries resulting from embers in poorly extinguished fires.^2^ Smothering campfires with sand is associated in postfire injuries due to its strong heat retaining properties that remain for up to 1 week, making the campfire dangerous even without evidence of flames. To further illustrate the danger of hot and buried coals, one of our burn surgeons constructed a backyard campfire to elucidate the heightened level of risk that embers or coals pose. Embers or hot coals can retain heat for up to 12 hours after being extinguished. A fire was started with one wheelbarrow of wood at 8:00 p.m. with the last wood added at around 10:30 p.m., with the temperature of a blazing fire to be above what the thermometer could detect. The subsequent morning at 8:00 a.m., the temperature of the coals was measured to be 148°C. Pine needles were then placed on the coals, which went on to ignite a fire. After the demonstration, the fire was appropriately extinguished with water.^2–4,9^

Utilization of healthcare resources in the treatment and management of these complications reinforces the need to investigate the issue further, and we seek to expand on the limited data. Cahill et al highlight the need for effective prevention strategies and need for education to reduce the amount of recreational fires.^3^ Furthermore, Hoang et al retrospectively reviewed the incidence of burns from recreational fires after a statewide ban in New Mexico and observed a significantly reduced amount of admissions.^10^ The findings in this study are in agreement with Hoang et al that prevention efforts should focus on age specific demographics, and local safety campaigns should educate families to stay more than 4 feet away from campfires.^10^ Collaboration with local fire departments to highlight guidelines and precautions with campfires and bonfires plays an important role in preventing burns.^10,11^ The harsh surgical and medical consequences of these burns in young children is a justification to further investigate these preventable injuries.^12–14^ Analyzing injury patterns and morbidity resulting from thermal burns is crucial to tailoring fire safety campaign to the public who may not otherwise recognize the dangerous implications these fires can pose to children.^15,16^ Shriner’s Burn Institute in 1996 reviewed series burns from campfires and outdoor cooking and subsequently initiated a safety campaign with 10,000 fliers distributed to regional camping facilities and communities.

It is a critical part of our agenda to initiate safety campaigns within our community similar to Shriner, which launched regional and statewide programs within schools in an effort to demonstrate safe behaviors when in the presence of a campfire. In our unit, social workers are commonly enlisted to explain precautions and safety for burned patients, but targeted educational pamphlets distributed at discharge could be more valuable. Our team therefore designed a flyer emphasizing campfire safety and reiterating national recommendations on safe burning. This brochure is being distributed to local fire departments, campgrounds, parks with fire pits and open grills, as well as clinic waiting rooms to highlight essentials on campfire safety. Given that in 64% of our patients the location/setting in which the injury occurred could not be identified efforts are being made to document the location/setting (home vs park or camp) in the electronic medical record so we can tailor our education to the patient’s family.

Air quality regulations in Michigan allow for the burning of logs, brush, charcoal, and materials for the purposes of recreation unless local county laws prohibit. Counties and townships have distinct guidelines for obtaining permits for open burning of paper, trees, leaves, and other sources that emit smoke and chemicals directly into the atmosphere. Most campfires do not require permits nor is it a requirement to follow open burning guidelines. Despite the lack of regulation, following the guidelines is encouraged for all fires. According to the Flint Fire Department, burn permits are issued the day the resident wishes to conduct open burning and are valid until midnight.^17^ They are only issued on the actual day of the burn to assess safe burning conditions such as wind speed, as burning is prohibited if winds exceed 10 miles per hour. Flint guidelines mandate that fires excluding barbecue grills should be at least 10 feet away from debris, buildings, combustible materials, or properties, and burn pits cannot exceed 3 feet high and 4 feet wide parameters.^17^ These regulations exclude barbecue, gas, and charcoal grills. Water sources in the form of extinguishers, garden hoses, or two 5-gallon buckets of water should be available for fire control as well as for use to extinguish the fire afterwards. Accelerants are not permitted to start fires; however, our results reflect the use of accelerants in 11% of injuries and one of the major culprits in burn injuries. State guidelines require constant supervision and attention from competent adults until the fire is extinguished and must be out by dark.^17^ It is recommended to check local city and township websites in order to find county specific education on safe construction as well as who to contact for assistance. In Michigan state parks, elevated and double walled pits are endorsed as safe options to construct bonfires.

## CONCLUSION

Burns secondary to recreational fires (camp or bonfires) are a cause of morbid injuries given that the hands are the most common injured sites. As already established in the literature, day old embers from inappropriately extinguished recreational fires are still dangerously hot and are able to cause significant thermal injuries. Campfire consciousness relies on schools, communities, and hospitals becoming involved in injury prevention efforts dedicated to educating children and parents before and throughout the peak summer season. Burn units have a unique opportunity to become involved through the use of fliers and campaigns, which highlights the safe behavior around campfires and bonfires.
